# Anti‐inflammatory treatment rescues memory deficits during aging in *nfkb1* ^−/−^ mice

**DOI:** 10.1111/acel.13188

**Published:** 2020-09-11

**Authors:** Edward Fielder, Clare Tweedy, Caroline Wilson, Fiona Oakley, Fiona E. N. LeBeau, João F. Passos, Derek A. Mann, Thomas von Zglinicki, Diana Jurk

**Affiliations:** ^1^ Biosciences Institute Ageing Research Laboratories Campus for Ageing and Vitality Newcastle University Newcastle upon Tyne UK; ^2^ Biosciences Institute Faculty of Medical Sciences Newcastle University Newcastle UK; ^3^ Bioscience Institute Immunity and Inflammation Newcastle Fibrosis Research Group Faculty of Medical Sciences Newcastle University Newcastle upon Tyne UK; ^4^ Robert and Arlene Kogod Center on Aging Mayo Clinic Rochester MN USA; ^5^ Department of Physiology and Biomedical Engineering Mayo Clinic Rochester MN USA

**Keywords:** aging, cognitive decline, hippocampus, memory, neuroinflammation, senescence

## Abstract

Chronic inflammation is a common feature of many age‐related conditions including neurodegenerative diseases such as Alzheimer's disease. Cellular senescence is a state of irreversible cell‐cycle arrest, thought to contribute to neurodegenerative diseases partially via induction of a chronic pro‐inflammatory phenotype. In this study, we used a mouse model of genetically enhanced NF‐κB activity (*nfκb1* ^−/−^), characterized by low‐grade chronic inflammation and premature aging, to investigate the impact of inflammaging on cognitive decline. We found that during aging, *nfkb1* ^−/−^ mice show an early onset of memory loss, combined with enhanced neuroinflammation and increased frequency of senescent cells in the hippocampus and cerebellum. Electrophysiological measurements in the hippocampus of *nfkb1* ^−/−^ mice *in vitro* revealed deficits in gamma frequency oscillations, which could explain the decline in memory capacity. Importantly, treatment with the nonsteroidal anti‐inflammatory drug (NASID) ibuprofen reduced neuroinflammation and senescent cell burden resulting in significant improvements in cognitive function and gamma frequency oscillations. These data support the hypothesis that chronic inflammation is a causal factor in the cognitive decline observed during aging.

## Graphical abstract

Chronic inflammation induces cellular senescence and leads to premature aging and cognitive decline. Anti‐inflammatory treatment reduces neuroinflammation and senescent cell burden, and ameliorates cognitive impairment.

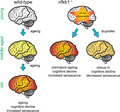

## 1 INTRODUCTION

Chronic inflammation is a feature of many neurodegenerative diseases and is associated with age‐dependent cognitive decline. Biomarkers of systemic inflammation are elevated in individuals with cognitive decline and have been hypothesized to contribute to higher risk of developing dementia (Bradburn, Sarginson, & Murgatroyd, 2018).

Cellular senescence is a state characterized by an irreversible cell‐cycle arrest and can be triggered by a wide range of stressors, including telomere dysfunction, oxidative stress, and inflammation among others (Gorgoulis et al., 2019). Senescence is accompanied by a host of phenotypic changes driven by several signaling pathways and maintained by a complex network of auto‐ and paracrine reinforcement (Ogrodnik, Salmonowicz, Jurk, & Passos, 2019). Senescence is characterized by the secretion of a number of pro‐inflammatory cytokines, chemokines, and matrix proteases, commonly named the senescence‐associated secretory phenotype (SASP), with many regulated through NF‐κB (Coppé et al., 2008). A chronic induction of the SASP is thought to contribute to age‐related pathology during aging and age‐related diseases, including neurodegenerative diseases such as Alzheimer's disease (AD) (Musi et al., 2018) and Parkinson's disease (PD) (Chinta et al., 2018). Accordingly, studies have shown that senescent cells increase in the brain during aging and neurodegenerative diseases (Jurk et al., 2012) and that removing senescent cells improves phenotypes in mouse models of PD (Chinta et al., 2018), AD, and tau‐dependent neurodegenerative diseases (Bussian et al., 2018; Musi et al., 2018; Zhang et al., 2019). However, the mechanisms driving cellular senescence in the brain *in vivo* are not elucidated.

We have previously reported that chronic inflammation is a driver of senescence and premature aging. We found that *nfkb1* ^−/−^ mice, which have increased inflammation due to the absence of the NF‐κB1 (p50) subunit, exhibit increased frequency of senescent cells and aging phenotypes in multiple organs and early mortality (Correia‐Melo et al., 2019; Jurk et al., 2014; Wilson et al., 2015). However, the impact of NF‐κB1 deletion on cognitive decline and senescent cell burden in the context of brain aging has not been fully investigated.

Here, we found that during aging, *nfkb1* ^−/−^ mice show an early onset of memory deficits, combined with neuroinflammation and increased frequency of senescent cells in the hippocampus and cerebellum. Extracellular field potential recordings in the hippocampus of *nfkb1* ^−/−^ mice revealed deficits in gamma frequency oscillations (20–80 Hz) consistent with impaired memory. Finally, treatment with the nonsteroidal anti‐inflammatory drug (NSAID) ibuprofen reduced inflammation and senescent cell burden and led to significant improvements in cognitive function and gamma frequency oscillations. Our findings suggest that chronic inflammation is a major driver of the cognitive decline observed during aging. Furthermore, our data indicate that under conditions of genetically enhanced NF‐κB activation, anti‐inflammatory treatment improves cognitive function and reduces senescence in the brain.

## 2 RESULTS

### 2.1 The anti‐inflammatory drug ibuprofen reduces neuroinflammation in a mouse model with enhanced NF‐κB activation

*Nfκb1* ^−/−^ mice exhibit chronic inflammation due to the absence of the NF‐κB1 (p50) subunit, which, as a p50:p50 homodimer, acts as a suppressor of NF‐κB activity by competing with RelA (the classic pro‐inflammatory NF‐κB subunit)‐containing dimers and by recruiting histone deacetylase 1 (HDAC1) leading to repression of transcription (Pereira & Oakley, 2008).

**FIGURE 1:**
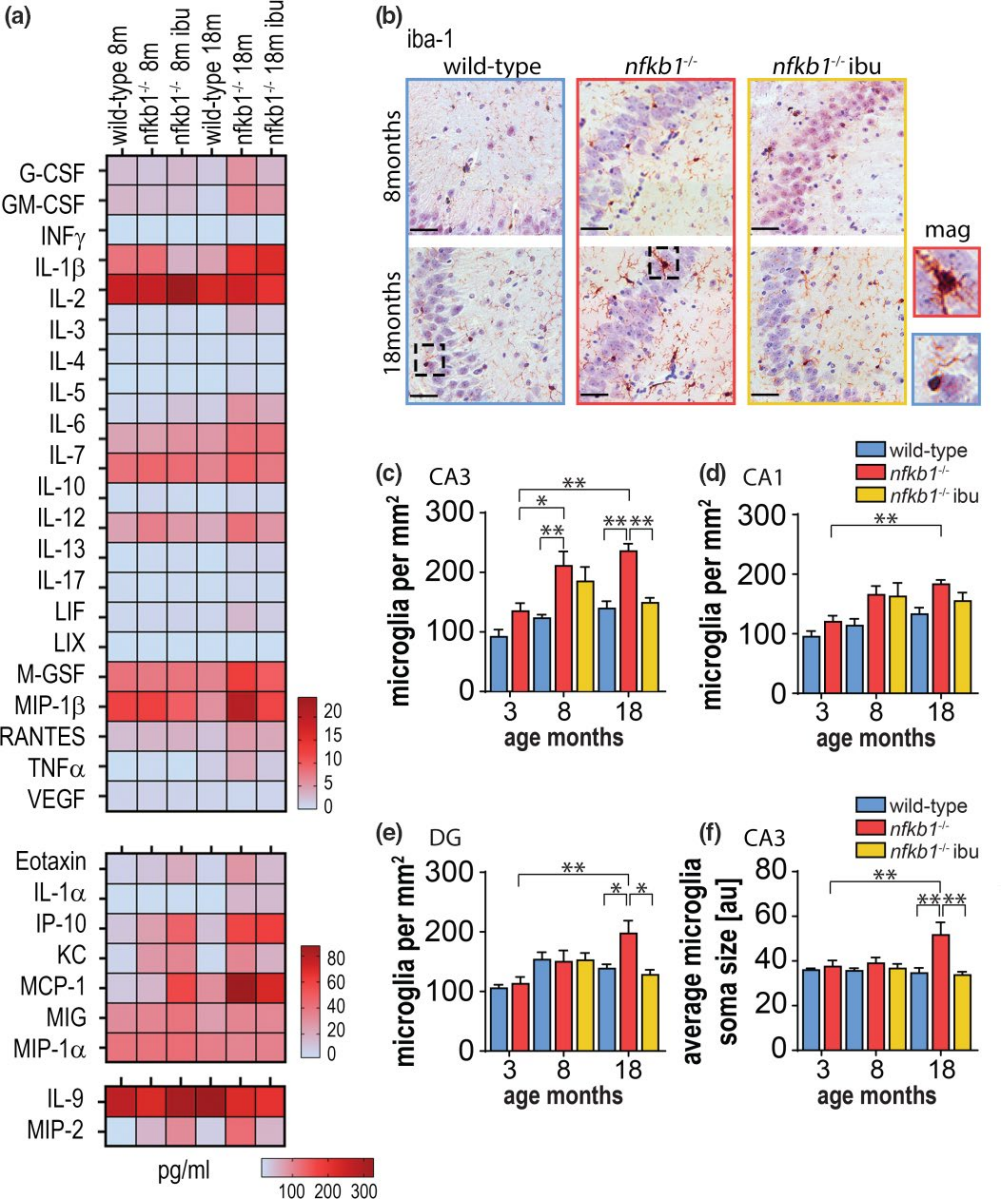
Treatment with NSAID reduces neuroinflammation in premature aging mouse model. At 6 and 9 months of age, male wild‐type and *nfkb1* ^−/−^ mice were split into groups and assigned to control or treatment groups before harvested at 8 and 18 months of age, respectively (see Figure S1a). (a) Cytokine expression pattern in whole brain homogenates for 8‐ and 18‐month‐old wild‐type and *nfkb1* ^−/−^ mice treated with/without ibuprofen. Data shown in pg/ml. (b) Micrographs showing iba‐1 IHC staining in hippocampus of paraffin‐embedded brain sections in 8‐ and 18‐month‐old animals in the indicated groups. Areas in dashed lines are shown magnified at the right, scale bars = 50 μm. Frequencies of microglia per mm^2^ hippocampus in (c) CA3, (d) CA1, and (e) DG of wild‐type and *nfkb1* ^−/−^ mice. (f) Average soma size per microglia was assessed in CA3 of the hippocampus. Significant differences (one‐way ANOVA) are indicated with *p < 0.05 and **p < 0.001. *n* = 4–5 mice per group

In order to study the effects of chronic inflammation on cognition, wild‐type and *nfkb1* ^−/−^ mice were aged. *nfkb1* ^−/−^ animals were given short‐term (for 2 months starting at 6 months of age) or long‐term (for 9 months starting at 9 months of age) treatment with ibuprofen (Figure S1a). To determine whether *nfkb1* ^−/−^ mice exhibited inflammation in the brain, a cytokine array was performed on whole brain homogenates (Figure 1a). We found that most cytokines increased more in *nfkb1* ^−/−^ animals, especially at higher age, and observed that the majority of chemokines and cytokines declined significantly after long‐term ibuprofen treatment (Figure 1a, Figure S1b‐g). Quantification of microglia, the resident immune cells of the brain, showed a significant increase in their numbers in the CA1, CA3 regions and dentate gyrus (DG) of the hippocampus proper (Figure 1b‐e). Further confirming the presence of enhanced neuroinflammation in *nfkb1* ^−/−^ mice, an increase in microglial soma size was observed, which is an indicator of microglia activation (Figure 1f). Importantly, we found that long‐term treatment with ibuprofen significantly reduced the number and soma size of microglia (Figure 1b‐f). Interestingly, if we correlate microglia soma size with expression of pro‐inflammatory cytokines in whole brain homogenates from 18‐month‐old wt or *nfkb*1^−/−^, we observed a positive linear correlation between microglia activation and expression of known SASP factors IL‐6, RANTES, MCP‐1, and IP‐10 (Figure S1i).

### 2.2 Chronic inflammation is associated with increased senescent cell burden, which is reduced by ibuprofen treatment

We have previously shown that *nfkb1* ^−/−^ mice acquire increased frequencies of senescent cells in the liver, intestine, and lung, which impacts on their regenerative capacity (Correia‐Melo et al., 2019; Jurk et al., 2014). In order to investigate whether *nfkb1* ^−/−^ mice show early onset of senescence in the brain, we evaluated various senescence markers in the hippocampus of these mice. We focused on the hippocampus due to its core role in memory function and cognition, and the functional, structural, and neurobiological changes it undergoes during aging (Bettio, Rajendran, & Gil‐Mohapel, 2017).

**FIGURE 2:**
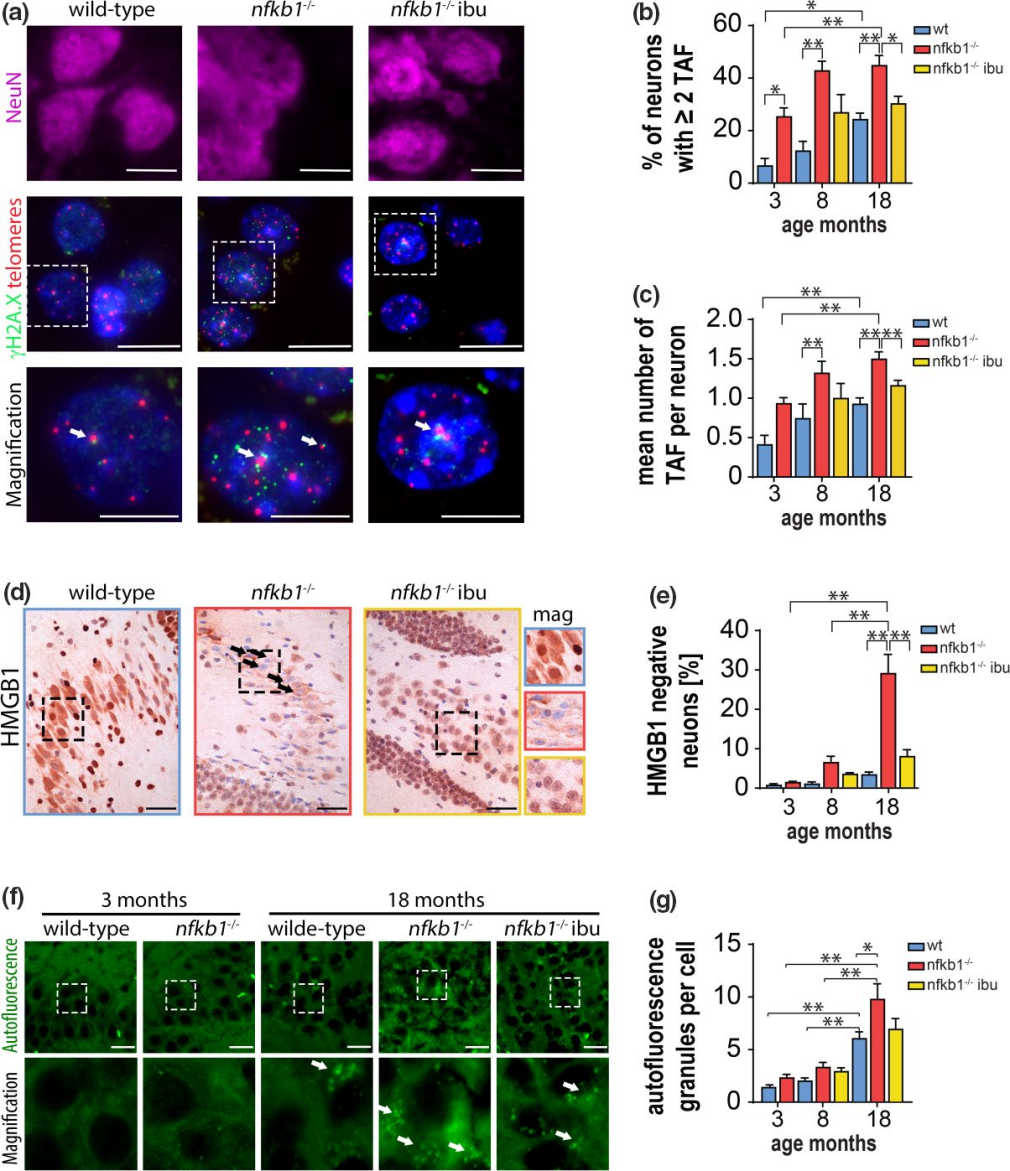
Chronic inflammation leads to neuronal senescence and can be ameliorated by NSAID treatment. (a) Representative images showing γ‐H2A.X (green) and telomere FISH (red) in NeuN (pink)‐positive cells of the indicated animals at 18 months of age. White arrows indicate telomere‐associated foci (TAF), and area in white dash‐lined quadrat is shown in magnification below. Scale bars = 20 μm in magnified images = 10 μm. (b) Percentage of neurons with ≥2 TAF and (c) mean number of TAF. (d) Representative images showing HMGB1 IHC staining in CA3 of the hippocampus in 18‐month‐old animals. Black arrows indicate neurons with loss of nuclear HMGB. Areas in dashed lines are shown magnified on right. Scale bars = 50 μm. (e) Percentage of neurons showing nuclear HMGB1 loss. (f) Micrographs showing lipofuscin granules (autofluorescence) in hippocampal neurons. White arrows indicate granules, and area in white dash‐lined quadrat is shown in magnification below. Scale bars = 20 μm. (g) Graph showing mean number of autofluorescent granules per neuron in indicated mice and treatments. Significant differences (one‐way ANOVA) are indicated with *p < 0.05 and **p < 0.001. *n* = 4–7 mice per group

Telomere dysfunction, characterized by the association of DNA damage response (DDR) proteins with telomeres, has also been shown to increase in multiple mammalian tissues during aging and age‐related diseases (Anderson et al., 2019; Birch et al., 2015; Hewitt et al., 2012) and is generally believed to be a robust marker of cellular senescence. In order to investigate whether telomere dysfunction was associated with neuronal senescence, we performed immunoFISH combining immunofluorescence against DNA damage response protein γ‐H2A.X, neuronal marker (NeuN), and *in situ* hybridization using a telomere‐specific PNA probe. We observed that while the number of γ‐H2A.X foci did not change significantly between groups, with age or with treatment (Figure S2a), telomere‐associated DNA damage foci (TAF) increased significantly with age in both genotype treatments (Figure 2a‐c). Furthermore, TAF was already significantly elevated in young *nfkb1* ^−/−^ in comparison with control animals, increased even further with age, and significantly reduced by ibuprofen treatment (Figure 2a‐c). Interestingly, by comparing the distribution of FISH intensities of telomeres that did not colocalize with γ‐H2A.X (non‐TAF) with those colocalizing with γ‐H2A.X (TAF) in neurons from aged wild‐type mice, we did not find those colocalized with γ‐H2A.X to be significantly shorter than those without, similar to findings in other murine tissues during aging such as liver (Hewitt et al., 2012), lung (Birch et al., 2015), heart (Anderson et al., 2019), and neurons from baboons (Fumagalli et al., 2012) (Figure S2b). This suggests that dysfunctional telomeres in neurons are not the result of reduced telomere length.

In order to investigate whether neurons expressed other markers of senescence, we performed immunohistochemistry for HMGB1, a protein that has been shown to be excluded from the nucleus in senescent cells (Davalos et al., 2013). We found that *nfkb1* ^−/−^ mice contained higher numbers of neurons displaying nuclear HMGB1 exclusion in comparison with wild‐type mice in the hippocampus. Furthermore, HMGB1 exclusion increased with age and was reduced by long‐term ibuprofen treatment (Figure 2d,e). Lipofuscin, a nondegradable aggregate of oxidized lipids, proteins, oligosaccharides, and transition metals, has been proposed as a marker of cellular senescence (Evangelou et al., 2017). We found that lipofuscin granules were significantly increased in neurons from old *nfkb1* ^−/−^ in comparison with wild‐type mice (Figure 2f,g). While neurons from *nfkb1* ^−/−^ mice treated with ibuprofen showed reduced numbers of lipofuscin granules, the difference was not statistically significant (Figure 2g). Further supporting an increased frequency of senescent cells in the hippocampus, we found increased numbers of p21‐positive neurons in 18‐month‐old *nfkb1* ^−/−^ mice and a reduction upon ibuprofen treatment (Figure S2c).

We have recently hypothesized that increased senescent burden may be a contributing factor to decreased neurogenesis (Ogrodnik, Zhu, et al., 2019). Consistent with this hypothesis, we found a decrease in DCX‐positive neurons in the dentate gyrus (DG) of *nfkb1* ^−/−^; however, we found no significant rescue upon ibuprofen treatment (Figure S2d).

We had previously reported that with age, Purkinje neurons in the cerebellum express senescence markers in wild‐type mice (Jurk et al., 2012). Analysis of senescence markers in Purkinje neurons in the cerebellum of *nfkb1* ^−/−^ mice showed similar patterns as seen in the hippocampus. *Nfkb1* ^−/−^ mice showed an age‐dependent increase in TAF but not in the total number of γ‐H2A.X foci when compared to wild‐type (Figure S2e,f). Additionally, *nfkb1* ^−/−^ mice exhibited increased senescence markers lipofuscin, p21 expression, and HMGB1 nuclear exclusion when compared to wild‐type mice, which were reduced upon ibuprofen treatment (Figure S2i‐m). Interestingly, we observed that HMGB1 nuclear exclusion was more prominent in granule cells in close proximity to HMGB1‐negative Purkinje neurons, supporting the idea that senescence may spread to neighboring cells or that senescence in Purkinje neurons may otherwise alter trophic signaling to granule cells (Nelson et al., 2012) (Figure S2n).

### 2.3 Reduced spatial discrimination and memory in *nfkb1* ^−/−^ mice is improved with ibuprofen

**FIGURE 3:**
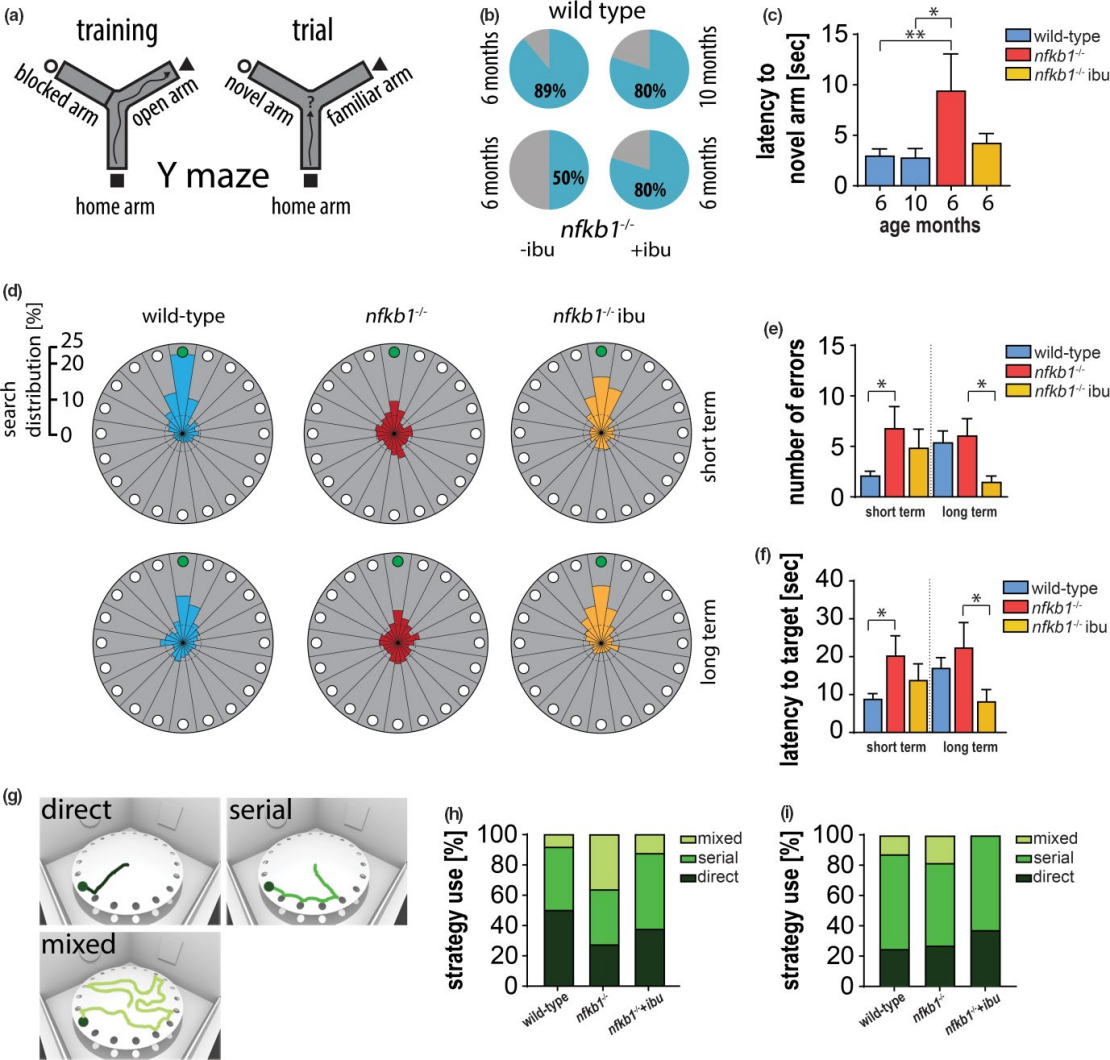
Reduced spatial discrimination and memory in *nfkb1* ^−/−^ mice can be rescued by NSAID. (a) Cartoon showing Y‐maze setup for training and trial period. (b) Pie‐charts depicting first choice of arm entry (blue = novel arm, gray = familiar arm) in Y‐maze. (c) Latency to enter novel arm. (d) Visual depiction of search distribution around the Barnes maze in 18‐month‐old animals (target hole in green at 12 o'clock position). Graphs showing (e) average number of errors prior and (f) latency to finding the target hole in 18‐month‐old animals. (g) Visual portrayal of search strategies in Barnes maze testing. Graphs displaying the percentage of each strategy used to find the target hole in (h) 8‐month and (i) 18‐month‐old animals. Significant differences (one‐way ANOVA, (e‐f) two‐way ANOVA) are indicated with *p < 0.05 and **p < 0.001. *n* = 4–18 mice per group in Y‐maze, 8–11 in Barnes maze

It has been hypothesized that inflammaging is a contributor to the cognitive decline observed during the aging process. In order to test this hypothesis, we first assessed short‐term memory and spatial learning using a forced alternation arm Y‐maze test (Figure 3a). In contrast to *nfkb1* ^−/−^ mice, wild‐type mice showed a preference for exploring the novel arm (Figure 3b). Treatment with ibuprofen for 2 months increased the frequency of primary exploration of the novel arm and significantly shortened the latency to novel arm entry (Figure 3b,c), this preference for the novel arm during exploration within the first 2 min of the test is indicated by a higher alternation index. *Nfkb1* ^−/−^ showed low alternation index, which was rescued by ibuprofen (Figure S3a).

To assess both short‐ and long‐term memory, we used the Barnes maze test, which measures spatial memory by training animals to find an escape box with the help of visual clues attached to the surrounding walls. We found that young wild‐type mice explored the target hole more frequently (up to 25%) than *nfkb1* ^−/−^ mice; however, 2‐month treatment with ibuprofen did not alter the search distribution significantly (Figure S3b). However, 8‐month treatment with ibuprofen significantly improved short‐ and long‐term memory in 18‐month‐old *nfkb1* ^−/−^ (Figure 3d‐f; Figure S3d).

Barnes maze testing allowed us to analyze in more detail the search strategies between groups. There are 3 different search strategies animals can present: (a) direct search (mouse directly searches target or adjacent hole), (b) serial search (mouse searches at least 2 sequential holes before finding the target hole), and (c) mixed search (mouse searches randomly) (Figure 3g). A mixed search strategy is generally an indicator of a more severe cognitive impairment. We found that at 18 months of age, 37% of *nfkb1* ^−/−^ mice applied the mixed/random search strategy in contrast to 8% of wild‐type mice during short‐term memory testing. Importantly, the percentage of *nfkb1* ^−/−^ mice applying this search strategy was reduced to 12.5% upon ibuprofen treatment (Figure 3h). Similar results were obtained during long‐term memory testing (Figure 3i).

### 2.4 Deficits in neuronal oscillations in *nfkb1* ^−/−^ mice are improved with ibuprofen

Neuronal oscillations are rhythmic changes in neuronal activity and occur at the level of individual neurons and in the synchronous firing of arrays of neurons (Gray, 1994). Neuronal network oscillations are seen throughout the entire mammalian brain and can bind the activity of arrays of neurons, often between multiple brain areas and hemispheres (Axmacher, Mormann, Fernández, Elger, & Fell, 2006; Singer, 1999). This allows the coordination of activity in a manner dependent on network connectivity, rather than physical proximity, and enables proper cognitive function (Axmacher et al., 2006; Singer, 1999). Gamma frequency is generated within the CA3 region and projects to the CA1 region (Csicsvari, Jamieson, Wise, & Buzsáki, 2003; Zemankovics, Veres, Oren, & Hájos, 2013). Neuronal activity within CA3 assists in the rapid acquisition of novel spatial information, pattern separation of similar spatial information, and the retrieval of information from place cells (Leutgeb, Leutgeb, Moser, & Moser, 2007; Montgomery & Buzsáki, 2007; Nakazawa et al., 2003). In “normal” aging, the CA3, unlike the CA1, undergoes a number of changes in excitatory properties and their ability to adjust firing to novel environments (Simkin et al., 2015; Wilson, Ikonen, Gallagher, Eichenbaum, & Tanila, 2005). Similarly, in aging C57Bl/6 mice reductions in gamma oscillations can be observed in the hippocampus by 16 months of age (Driver et al., 2007; Vreugdenhil & Toescu, 2005). Alterations to gamma oscillations can be seen in multiple neurological disorders and neurodegenerative conditions such as Alzheimer's disease, and are believed to play a significant role in the cognitive dysfunction associated with these diseases (Driver et al., 2007; Herrmann & Demiralp, 2005).

**FIGURE 4:**
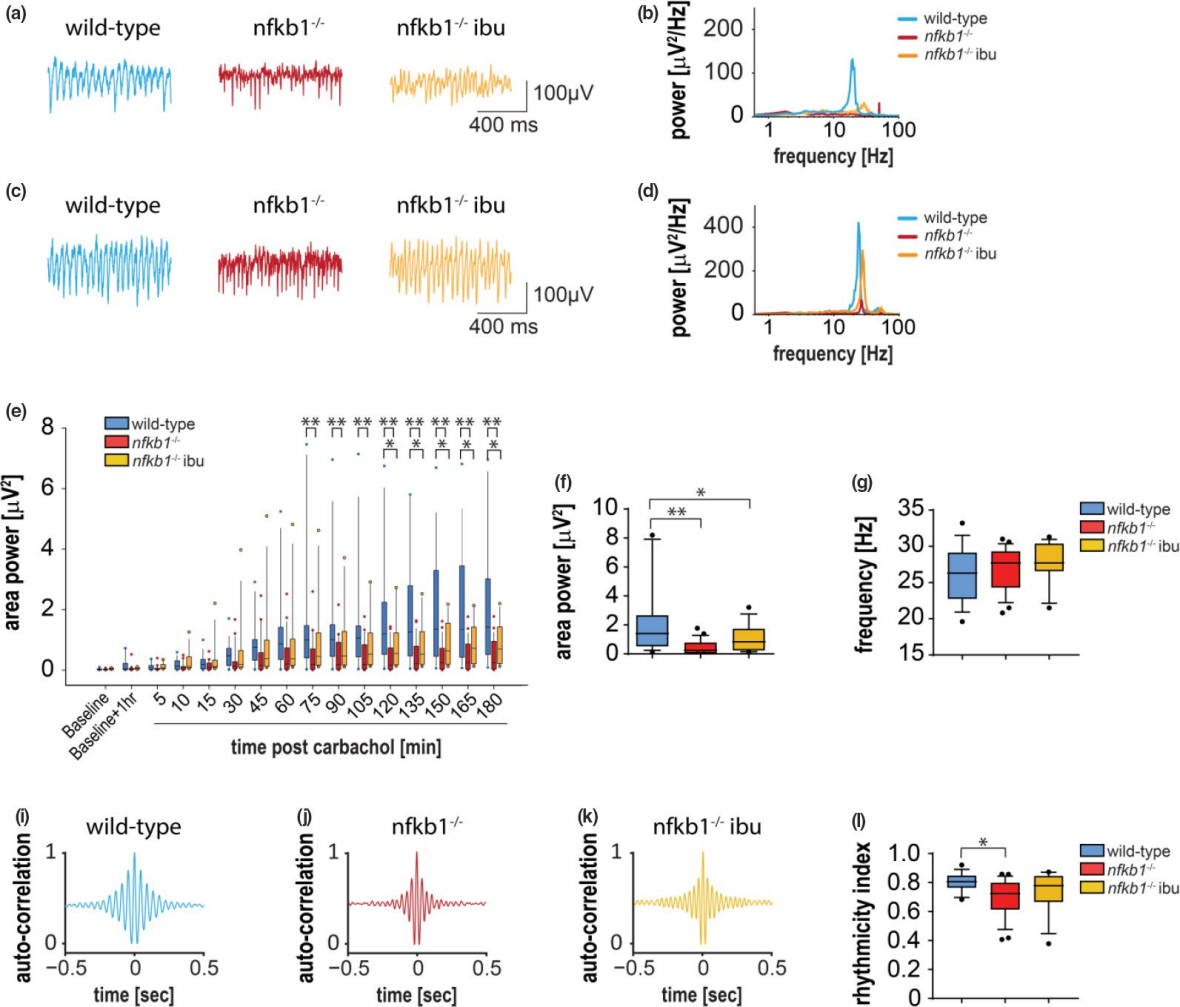
Deficits in neuronal oscillation is improved after NSAID treatment. (a) 1‐s example traces 30 min after carbachol and (b) at stable time‐point (c, d) shows the corresponding power spectra for 60‐s traces at 30 and 3 hr postcarbachol. (e) Box plot shows gamma frequency area power of the oscillations over time after carbachol application. Slices were allowed 1 hr in the recording chamber (baseline + 1 hr) before the addition of carbachol. Graphs showing (f) oscillation area and (g) oscillation frequency area power at stable time‐point. (h) Representative autocorrelations of gamma oscillations recorded in CA3. (i) Normalized rhythmicity index (RI) of gamma frequency oscillations in CA3. Significant differences (two‐way repeated measures for e‐g and one‐way ANOVA for (i) are indicated with *p < 0.05 and **p < 0.001. *n* = 3–6 slices per mouse and 4–5 mice per group

To assess whether *nfkb1* ^−/−^ mice showed alterations in network oscillations, we conducted electrophysiological measurements in hippocampal slices of wild‐type and *nfkb1* ^−/−^ mice with or without ibuprofen treatment at 8‐9 months of age. Carbachol, a cholinergic agonist, was bath‐applied to evoke a low gamma frequency (~20‐35 Hz) neuronal oscillations recorded in the CA3 region (Fisahn, Pike, Buhl, & Paulsen, 1998; Zemankovics et al., 2013). After addition of carbachol, the area power of gamma frequency oscillations gradually increased, stabilizing at 3–4 hr postcarbachol administration (Figure 4a‐g). We observed that gamma frequency oscillations in *nfkb1* ^−/−^ mice had a significantly smaller area power than wild‐type mice. Treatment with ibuprofen showed a trend toward stronger gamma oscillations, with gamma frequency power intermediate between the values of wild‐type and untreated *nfkb1* ^−/−^ slices (Figure 4a‐d). *Nfkb1* ^−/−^ gamma oscillation area power was significantly lower than in slices from wild‐type mice 75 min postcarbachol administration onward, while *nfkb1* ^−/−^ that had been treated with ibuprofen did not diverge from wild‐type values until 2 hr after carbachol (Figure 4e). Once gamma oscillations had stabilized, *nfkb1* ^−/−^ brains showed significantly smaller area powers, but no change was observed in oscillation frequency (Figure 4f,g).

The generation of gamma frequency oscillations relies on reciprocal interactions between pyramidal and interneurons, and requires the precise timing of firing to generate a synchronous oscillation (Whittington, Cunningham, LeBeau, Racca, & Traub, 2011). As the data suggested impairments in the generation of gamma frequency oscillations in the CA3 *stratum radiatum* of *nfkb1* ^−/−^ mice, the rhythmicity of these oscillations was examined. Autocorrelations of recordings from CA3 taken during oscillations were compared between wild‐type, *nfkb1* ^−/−^, and ibuprofen‐treated *nfkb1* ^−/−^ mice (Figure 4h,i). Gamma frequency oscillations in slices from wild‐type mice showed high rhythmicity index scores, indicating a synchronous gamma frequency oscillation is being generated; untreated *nfkb1* ^−/−^ slices show a significant reduction in the rhythmicity of gamma frequency oscillations compared with wild‐type slices; however, gamma frequency oscillations in those treated with ibuprofen were improved and did not significantly differ in comparison with slices from wild‐type animals (Figure 4i).

## 3 DISCUSSION

Aging is a major risk factor for the development of neurodegenerative diseases (Hou et al., 2019) and has been associated with a low‐grade sterile inflammation, commonly termed inflammaging (Franceschi et al., 2007). Furthermore, inflammaging has been associated with deficits in cognitive function (Barrientos, Kitt, Watkins, & Maier, 2015; Weaver et al., 2002). Several studies involving mice suggest that increased chronic systemic inflammation may be a driving factor in age‐dependent cognitive decline. Parabiosis experiments, in which young and aged mice are sutured together and share the same circulation, show that exposure to the bloodstream of aged mice leads to significant cognitive impairments in young mice (Villeda et al., 2011). Genetic interventions targeting pathways involved in the regulation of inflammation have also been shown to impact on age‐related functional decline; the deletion of Nlrp3, a major component of the inflammasome complex, has been shown to improve cognitive function and reduce expression of pro‐inflammatory cytokines in the brain (Youm et al., 2013). Breakdown in the blood–brain barrier, which is a common feature of aging and neurological disorders, has been recently shown to trigger TGF‐β signaling in astrocytes and induce cognitive impairments. Importantly, inhibition of TGF‐β in aged mice reversed the detrimental phenotypes (Senatorov et al., 2019). While these studies suggest a causal relationship between inflammaging and cognitive decline, the underlying mechanisms are still poorly elucidated.

One possible mechanism by which inflammaging can contribute to the development of neurodegenerative diseases is via the induction of cellular senescence. Clearance of senescent cells whether genetically or pharmacologically has been shown to improve phenotypes in mouse models of Parkinson's disease (Chinta et al., 2018), tau‐dependent neurodegenerative diseases (Bussian et al., 2018; Musi et al., 2018; Zhang et al., 2019), and neuropsychiatric disorders (Ogrodnik, Zhu, et al., 2019). Mechanistically, it is still unclear how senescence impacts on neurodegeneration; however, it has been speculated that this is partially mediated via the SASP, which can spread senescence to surrounding cells (Acosta et al., 2013; Xu et al., 2018), induce neuronal cell death (Turnquist et al., 2016), and negatively impact on neurogenesis (Chinta et al., 2018; Ogrodnik, Zhu, et al., 2019). Consistent with this hypothesis, we found that brains from *nfkb1* ^−/−^ mice show increased frequency of senescent cells and reduced markers of neurogenesis, which is consistent with a previous report (Denis‐Donini et al., 2008). In our study, we focused mostly on neurons in the hippocampus, given its important role in memory formation; however, a more thorough characterization of senescence in different cell types and brain regions is warranted. The hippocampus undergoes numerous changes during aging, including increased oxidative stress, neuroinflammation, and changes in gene expression, together with reduced neurogenesis and alterations in neuronal function (Bettio et al., 2017). So far, markers of senescence have been identified in neurons (Fielder, von Zglinicki, & Jurk, 2017; Jurk et al., 2012; Musi et al., 2018; Ogrodnik, Zhu, et al., 2019), astrocytes (Chinta et al., 2018; Ogrodnik, Zhu, et al., 2019), microglia (Bussian et al., 2018), and oligodendrocytes (Zhang et al., 2019) during aging and neurodegenerative diseases. Yet, the relative contribution of senescence in each of the cell types or different regions of the brain is still not understood.

Our findings indicate that long‐term treatment with ibuprofen reduces markers of senescence in neurons and brain inflammation, in a model with genetically induced chronic inflammation, suggesting that inflammation is a driver of senescence in the brain. Ibuprofen, through inhibition of cyclooxygenase enzyme COX‐2, could potentially reduce the paracrine induction of senescence by SASP factors (Acosta et al., 2013; Nelson et al., 2012). Additionally, while SASP factors, such as IL‐8, can promote the intracellular accumulation of ROS via CXCR2 (Acosta et al., 2008), ibuprofen also appears to directly impact on pathways contributing to aberrant production of reactive oxygen species (ROS) (Jurk et al., 2014), which can drive telomere dysfunction and senescence (von Zglinicki, 2002). It should be taken into account that short‐term treatment with ibuprofen can reduce senescence in other tissues (Jurk et al., 2014). It is therefore difficult to ascertain whether the observed improvements in cognition due to ibuprofen are the result of *in situ* effects on inflammation and senescence in the central nervous system or in the periphery. We cannot infer from our results the mechanisms by which ibuprofen is targeting senescent cells. We assume that the most likely scenario is that ibuprofen is either preventing the onset of senescence or acting as a senomorphic, reducing the expression of senescence markers without inducing cell death as we have shown previously (Jurk et al., 2014). However, it remains possible that ibuprofen may be contributing to the selected elimination senescent cells, whether directly by apoptosis or via the immune system.

Our data are consistent with other studies supporting a beneficial role for NSAIDs during normal aging in preclinical models. For instance, administration of COX inhibitor celecoxib in 12‐month‐old rats reduced age‐dependent increases in pro‐inflammatory cytokines in the hippocampus and improved performance in the Morris water maze hippocampus (Casolini, Catalani, Zuena, & Angelucci, 2002). Administration of COX2 inhibitors nimesulide, rofecoxib, and naproxen to 16‐month‐old mice improved passive avoidance performance and elevated plus maze (Jain, Patil, Kulkarni, & Singh, 2002). NSAID sulindac was shown to reduce neuroinflammation in the hippocampus and improve cognitive performance in 18‐month‐old rats (Mesches et al., 2004). Interestingly, we observed that short‐term treatment with ibuprofen increased the expression of a few pro‐inflammatory cytokines (albeit only MCP‐1 was significantly changed). There have been reports that NSAIDs can exacerbate inflammatory responses in certain contexts. One possible explanation is that this may be the outcome of NSAIDs causing injury to the gastrointestinal system and lead to leakage of commensal bacteria and/or LPS into the circulation provoking a systemic inflammatory response (Tugendreich, Pearson, Sagartz, Jarnagin, & Kolaja, 2006).

While our study indicates a potential role for NSAIDs as interventions against age‐dependent cognitive decline in the presence of inflammation, it should be noted that although inflammation is a major pathophysiological feature of most neurodegenerative disorders, anti‐inflammatory interventions have yielded mixed results in the clinic. NSAIDs are largely ineffective in the context of AD (Miguel‐Álvarez et al., 2015); however, some data indicate that ibuprofen may reduce the risk of developing conditions such as PD (Rees et al., 2011). As such, any effects of NSAIDs, such as ibuprofen, will undoubtedly be context‐dependent. Furthermore, more targeted interventions to reduce inflammation will be required, particularly since NSAIDs when taken inappropriately can be potentially dangerous and cause adverse effects (Bateman, 1994).

In conclusion, our data support the hypothesis that chronic inflammation is a causal factor in cognitive decline during aging and is associated with increased cellular senescence in the brain.

## 4 MATERIALS AND METHODS

### 4.1 Animals

Experiments were performed on male *nfkb1* ^−/−^ mice on a C57Bl/6 background and pure C57Bl/6 wild‐type controls. Pure background C57Bl/6 mice were a gift from Jorge Caamano (Birmingham University, UK). Mice were housed at the pathogen‐fee barrier area of the Newcastle Animal House (Centre for Comparative Biology). Mice were observed regularly and euthanized when they showed tumors or morbidity, in accordance with the Guidelines for Humane Endpoints for Animals Used in Biomedical Research. *Nfkb1* ^−/−^ mice were genotyped by our collaborators according the Jackson laboratory protocol for *nfkb1* ^−/−^ mice. Each mouse was weighed and measured once a week. Mice were housed in cages (56 × 38 × 18 cm, North Kent Plastics) of groups of 4–6 that did not change from weaning. Mice were provided with saw dust and paper bedding and had adlib access to water. Mice were housed at 20 ± 2°C under a 12‐hr light/12‐hr dark photoperiod with lights on at 07:00 hr.

For 8 month time‐point mice, mice received ibuprofen via a mini‐pump (mini‐osmotic pump, Alzet, model 2004) for a period of 2 months starting at 6 months of age. Ibuprofen was dissolved in PEG and DMSO (50:50) to a daily dosage of 50 mg per kg. A small incision was made on the right flank and a mini‐pump was inserted subcutaneously, and the wound was repaired with 7‐mm clips. After 28 days, a replacement was implanted. Under general anesthesia, pumps were surgically removed and a wound repair was performed. A small incision was made on the left flank and a new mini‐pump was inserted subcutaneously, and the wound was repaired with 7‐mm clips. Mice at 18 months and those used for electrophysiology were given ibuprofen mixed in soaked chow diet to a daily dosage of 50 mg per kg (mouse) per day. For 18 month time‐point mice, treatment was started at 9 months of age. In mice used for electrophysiology, treatment started at 3 months of age, till termination at 8 months.

Ethical approval was granted by the LERC Newcastle University, UK. All experiments were undertaken in compliance with UK Home Office legislation under the Animals (Scientific Procedures) Act 1986. Organs were either fixed in 4% paraformaldehyde or frozen in liquid nitrogen. Fixed tissues were processed and embedded in paraffin. All sections were cut at a thickness of 3 μm or 10 μm.

### 4.2 Immunohistochemistry

Tissues were deparaffinized and hydrated using two washes of Histoclear, followed by step‐down series of ethanol (100%, 90%, 70%) and then twice in distilled H_2_O, each step being 5 min per wash. Antigen retrieval was performed by incubation in 0.01 M Citrate Buffer, microwaved for 4 min at 800 W, then 10 at 450 W, and allowed to cool to room temperature, before being washed in water.

Endogenous peroxidase activity was blocked with 0.9% H_2_O_2_ for 30 min at RT. Samples were transferred to phosphate‐buffered saline (PBS) and drawn around with an immunopen. Blocking of nonspecific binding was performed with 70 μl per section of freshly prepared normal goat serum (NGS) 1:60 in 0.1% bovine serum albumin (BSA)/ PBS, for 30 min at room temperature in a humidified chamber (used for all subsequent incubations). This was followed by Avidin–Biotin blocking (each for 15 min, followed by PBS wash). Sections were then incubated overnight with primary antibody diluted in NGS/BSA/PBS at 4°C.

Sections were washed in PBS 3 times and incubated with biotinylated secondary anti‐mouse goat antibody in NGS/BSA/PBS for 30 min, then washed 3 times in PBS. ABC complex was prepared to manufacturer's instructions 30 min prior to use. Sections were washed 3 times in PBS and incubated in freshly prepared NovaRed solution prepared to manufacturer's instructions.

Sections were then rinsed in distilled H_2_O, placed in hematoxylin to counterstain, rinsed thrice in distilled H_2_O, placed in hot nondistilled H_2_O for 30 s, and then washed in distilled H_2_O. Sections were dehydrated in 2 × 30 s 95% ethanol, 2 × 30 s 100% ethanol, and 2 × 5 min Histoclear and mounted with DPX.

For p21 staining, antigen retrieval time at 800 W was increased to 30 min to increase antigen unmasking. For Iba1 and DCX stainings, the following alterations were made. 10‐μm paraffin‐embedded sections were used, rather than 3‐μm sections. Antigen retrieval was performed by incubation in 0.01 M Citrate Buffer heated by 4 min at 800 W, then 6 min at 450 W, and allowed to cool at room temperature. This reduced separation of sample from the slide while preserving good signal‐to‐noise ratio. For goat primary antibodies, blocking solution used for blocking, primary, and secondary antibody was freshly prepared 5% normal serum (of the secondary biotinylated antibody species) in PBS, for 30 min at room temperature. For Iba1, this was 5% normal horse serum in PBS.

Microscopy for Immunohistochemistry was performed with a Nikon Eclipse E‐800 Brightfield Camera. Tile‐scan images from the E‐800 were stitched using Microsoft Image Composite editor, if required. For analysis of iba1^+^ cells, a Leica DMi‐8 in Brightfield mode was used for a ×40 tile scan of the hippocampus. This was split into CA3, CA1, and DG. The number of iba1^+^ in each area and its size was recorded. The final count was expressed as number of iba1^+^ cells per mm^2^ in a 10 μm slice. DCX^+^ cells were counted along the DG blade, and the length of the DG blade at the subgranular zone was recorded in mm. Final count was expressed as number of DCX^+^ cells per mm. For the wild‐type and *nfkb1* ^−/−^ study, p21 immunohistochemistry was counted as positive for strong nuclear staining, or moderate to no staining. Neurons were identified by shape. HMGB1 cells were quantified as positive or negative. These were expressed as a percentage of cells counted.

### 4.3 Telomere ImmunoFISH

Deparaffinization and hydration were performed as described earlier. Sections were blocked with Mouse on Mouse Kit (Vector UK, PK‐2200) as per manufacture instructions, and incubated for 1 h with 1:500 NeuN antibody (Mouse IgG, Abcam, ab104224) with secondary 1:1000 Alexa Fluor 647 anti‐mouse (Goat IgG, Abcam, ab150119) for 30 min, both in MOM diluent. Following PBS washes, nonspecific blocking was performed for 30 min at room temperature with NGS/BSA/PBS, as well as Avidin–Biotin blocking (each for 15 min, followed by PBS wash). Sections were incubated with 1:250 primary rabbit γ‐H2A.X antibody (Cell Signaling) in NGS/BSA/PBS overnight at 4°C. Sections were washed with PBS 3 times, and incubated with 1:200 biotinylated secondary goat anti‐rabbit antibody (Vector Laboratories) in NGS/BSA/PBS for 30 min at room temperature. Following PBS wash, Avidin DCS (Vector Laboratories, 1:500 in PBS) was applied to the sections for 30 min, then washed again in PBS. Cross‐linking was performed using 4% paraformaldehyde/PBS for 20 min. Sections were washed in PBS and dehydrated using sequential 3‐min ice‐cold EtOH ethanol washes (70%, 90%, 100%) and then allowed to air‐dry. 10 μl of hybridization mix (2.5 μl 1 M Tris pH 7.2, 21.4 μl magnesium chloride buffer, 175 μl deionised formamide, 1 μl Cy‐3 CCCTAA peptide nucleic acid probe (Applied Biosystems), 12.5 μl Blocking Buffer (1:9 Roche Blocking reagent in autoclaved malic acid), and 33.6 μl deionised H_2_O) was applied and a coverslip placed upon the slide, after which DNA denaturation was performed by placing sections in an 80°C oven for 10 min. Sections were placed in humidified chamber for 2 hr at room temperature. Sections were then washed in 70% formamide/2 × SSC for 10 min, 2 × SSC 10 min, PBS 10 min on a mixing tray. Sections were mounted using VectorShield with DAPI (Vector Laboratories).

Microscopy for immunofluorescent stainings was performed with a wide‐field fluorescent microscope: a Leica DM‐5500‐B, fitted with a Leica DFC‐360‐FX camera and attached to a desktop running Leica LASAF‐2.1.0. Focal plane selection in fluorescence was selected using the DAPI channel, with z‐stack bounds based on telomere probe focus. Images were stored as unaltered.lif for later measurement. Measurement of number of γH2A.X foci per cell, and number colocalizing with telomeres was conducted under blinded conditions, with both the age and genotype of the animal masked. Neurons in the hippocampus were differentiated from glial cells based on location, morphology in the DAPI channel, and positive staining for the neuronal Abcam NeuN antibody, and Purkinje neurons based on location, morphology, and low intensity in the DAPI channel within the cerebellum (as these cells do not express NeuN).

### 4.4 Autofluorescence

Autofluorescence measurement was performed on paraffin‐embedded slides from the brains of wild‐type and *nfkb1* ^−/−^ knockout mice. Unprocessed slides were used for Purkinje cells, due to their large soma. The smaller size of hippocampal neurons necessitated the use of dewaxed and mounted slides to discern individual foci. Microscopy imaging of the slides was performed, using identical settings for lamp intensity, gain, lens aperture, and f‐stop between slides, with single image capture, at ×20 magnification for Purkinje neurons and A4 filter cube (band pass 360/40 nm, barrier filter band pass 470/40 nm), and ×40 for hippocampal neurons. Imaging was performed in equivalent morphological locations within the areas of interest between slides.

Measurement was performed blinded of age and genotype; in hippocampal neurons, mean number of autofluorescent foci was counted per cell. In Purkinje neurons, average image fluorescence was measured using a “Region of Interest” tool to select cells of interest in Fiji (ImageJ derivative). Signal increases linearly with slide and wax thickness, small differences between sittings can occur due to differing lamp intensity even if exposure is kept standard. To account for this, the signal:noise ratio is used. Noise, or “Background,” correction is performed by taking multiple readings from the edge of the tissue, where only paraffin is present, and dividing the signal by this value to obtain the ratio.

### 4.5 Cytokine/chemokine array

Brain tissue was frozen in liquid nitrogen at the time of harvest, stored at −80°C, and ground manually to a fine powder using a steel mortar and pestle. This was wiped clean between samples with 70% EtOH and cooled in liquid nitrogen. Powdered sample was stored at −80°C till homogenate preparation. 0.5 ml Homogenate Tris Buffer was added to powdered sample in a ceramic mortar and pestle, then cooled in liquid nitrogen. They were manually ground together, and mixed until they formed a paste, then liquid. This was spun for 30 min at 21.1 *g*. Supernatant was taken and stored at −80°C. The concentration was adjusted to 5 μg protein/ml and shipped to Eve Technologies Corporation, Calgary, Canada, for mouse cytokine/chemokine array MD31 using a Multiplexing LASER Bead Assay.

### 4.6 Electrophysiology

Animals were anesthetized with inhaled isoflurane prior to intramuscular injection of ketamine (≥100 mg/kg) and xylazine (≥10 mg/kg). When all responses to noxious stimuli had ceased, the mice were intracardially perfused with ~25 ml Modified Artificial CerebroSpinal Fluid (ACSF) [10 mM glucose, 252 mM sucrose, 24 mM NAHCO_3_, 3 mM KCl, 2 mM CaCl_2_, 2 mM MgSO_4_, 1.25 mM NaH_2_PO_4_]. Once perfused, the brain was removed and cerebellum and brainstem separated and stored in 4% PFA. The brain was sectioned using a Leica VT1000s into 450‐μM sections. These sections were trimmed to isolate the hippocampus and transferred to a holding chamber where they rested for 1 hr at ~33°C, supplied by ACSF, and humidified at 95% O_2_/5% CO_2_.

For extracellular recordings, electrodes are filled with ACSF (resistance 2–5 MΩ). Recordings for gamma frequency oscillation area power build‐up were taken from the CA3 region in the *stratum radiatum*. Administrating carbachol, a cholinergic agonist, to hippocampal slices stimulates synchronous low gamma frequency neuronal oscillations originating in CA3 and propagating to CA1 *stratum radiatum* (Fisahn et al., 1998; Traub et al., 2000). These oscillations persist for a number of hours, and in healthy slices will build‐up in area power and amplitude over time before stabilizing at approximately 3‐4 hr postcarbachol administration. Carbachol was administered into the bath solution, and 60‐s recordings were taken at set intervals for 3 hr, and then until gamma oscillations stabilized (defined as 3 successive recordings at 10‐ to 15‐min intervals with area power within 10% of each other, or after 3 hr postcarbachol administration).

Recordings were taken with an Axoclamp‐2B amplifier (Axon Instruments Inc.). Extracellular recordings were filtered at 0.001–0.4 kHz using Bessel filters. Noise from the mains electricity was subtracted from the signal using a Humbug (Digitimer). Data were re‐digitized at 10 kHz using an ITC‐16 interface (Digitimer). Data were recorded and analyzed using Axograph software (Axon Instruments Inc.).

60‐s recordings of activity were passed through a fast Fourier transformation in Axograph to provide power spectra. Area power for slow gamma oscillations was defined as area under the curve between 15 and 45 Hz. Frequency was defined at the peak of the oscillation. Oscillations were determined as stable at or after 3 hr. Oscillation rhythmicity index (RI) was measured from autocorrelations performed in MATLAB 2012, where the peak was normalized to 1.

Data were analyzed in Sigmaplot v12.5. *p* of <0.05 is taken as significant. Build‐up data were assessed with 2‐way repeated‐measures analysis of variance, with time and group (wild‐type, *nfkb1* ^−/−^ untreated, *nfkb1* ^−/−^ treated) as factors. Differences between genotype and treatment were assessed by 2‐way ANOVA without interactions. If the *p*‐value indicated a significant difference, a Holm–Sidak post hoc test was used to determine which parameters likely varied.

### 4.7 Barnes maze

The Barnes maze consists of an open circular surface (diameter = 92 cm), with a series of 20 holes (diameter = 5 cm) along the border and four visual markers on adjacent walls at 0, 90, 180, and 270° positions (cross, triangle, circle, rectangle). Visual icons are marked on the walls around the maze, and the maze is elevated so that mice cannot exit via the sides. Under one of these holes is attached the “target box,” which the mouse can enter and provides refuge from the bright and exposed open surface top. The experiment requires the subject to learn the spatial position of this escape box relative to a number of visual cues positioned on the surrounding walls. The mouse is placed in an opaque pipe (diameter = 11 cm, height = 15 cm), in the centre of the maze and allowed 10 s to settle. The pipe is removed and the timer started. An overhead camera, linked to a DVD recorder and monitor, allows the performance of the mouse to be observed and recorded.

Each mouse is assigned to one of the four visual markers, under which the escape box will be placed during training. In the initial run, naïve mice are guided to the escape hole and allowed to rest inside for 2 min to acclimatize. The “Acquisition phase” follows this, with mice running 4 trials per day, for 4 days. In each trial, the mouse is given 3 min to find and enter the escape hole. If the mouse does not find the hole in the allotted time, then it is again guided to the escape hole. The mouse is allowed to rest in the target box for 1 min. Between trials, the board and escape box are wiped with 70% EtOH to remove odor trails.

Mice do not always enter the target hole after finding it and may proceed to either further explore the maze or sit at the entrance hole. To account for this, measurements were taken of the strategy, time, and number of errors till first finding the target hole, these are referred to as the “primary” parameters. Primary errors, total errors, primary latency to find hole, total latency to enter hole, and search strategy were recorded. Errors were defined as head deflection and nose‐pokes into “false holes.” Search strategy was defined as (a) direct—moving directly to the target hole, or an adjacent hole prior to entry; (b) serial—finding the target hole after visiting at least two adjacent holes moving round the maze in a clockwise or counterclockwise fashion; and (c) mixed—search pattern involved moving across the center of the board or seemingly random selection of search areas.

Short‐term memory was tested on the fifth day in a 90‐s trial, without the target box present. Long‐term memory retention is tested in the same fashion on the twelfth day with no training between these trials. Location and frequency of visited holes, site of initial visit, and time spent in target quadrant were additionally recorded in short‐ and long‐term tests.

### 4.8 Forced alternation Y‐maze

A forced alternation Y‐maze setup was used. Each arm was 40 cm long, 8 cm wide, and 15 cm high. The end of each arm was marked with a black icon. The home cage was moved into the room for 1 hr prior to testing to allow mice to acclimatize.

In acquisition, one of the arms was blocked off by a white barrier. The mouse is placed in the home arm (+) and given 10 min to explore the two open arms of the maze (home and familiar). After this, the mouse was returned to the home cage for a 1‐hr inter‐trial period. Between runs, the maze was cleaned thoroughly with 70% EtOH between trials to remove odors. Up to 5 mice could be tested at a time.

After 1 hr, the mouse was placed in the home arm, with all 3 arms open and allowed to explore for 2 min. If the mouse climbs out of the maze, it was returned to the abandoned spot. Latency to novel arm, primary arm choice, and time spent in each arm were recorded. Arm discrimination index was scored as the time spent in the novel arm, divided by the total time spent in both the familiar and novel arms.

## CONFLICT OF INTEREST

F.O. and D.A.M. are directors and shareholders of Fibrofind limited.

## AUTHOR CONTRIBUTIONS

E.F. performed the majority of experiments and gathered data; C.T. and C.W. performed and evaluated individual experiments; D.J. with help from J.F.P., F.O., and T.v.Z. designed and supervised the study; and D.J. prepared figures and wrote the manuscript with contributions from D.A.M., F.O., E.F., F.E.N.L., T.v.Z., and J.F.P.

## Supporting information

Fig S1‐S3

Fig S1‐S3‐Legends

## Data Availability

The data that support the findings of this study are available from the corresponding author upon reasonable request.
