# Supplementary figures and images for: Anti‐inflammatory treatment rescues memory deficits during aging in nfkb1 −/− mice
Source: Aging Cell. 2020 Sep 11;19(10):e13188. doi: 10.1111/acel.13188 (PMC7576267; doi:10.1111/acel.13188)

Supplementary Figure 1

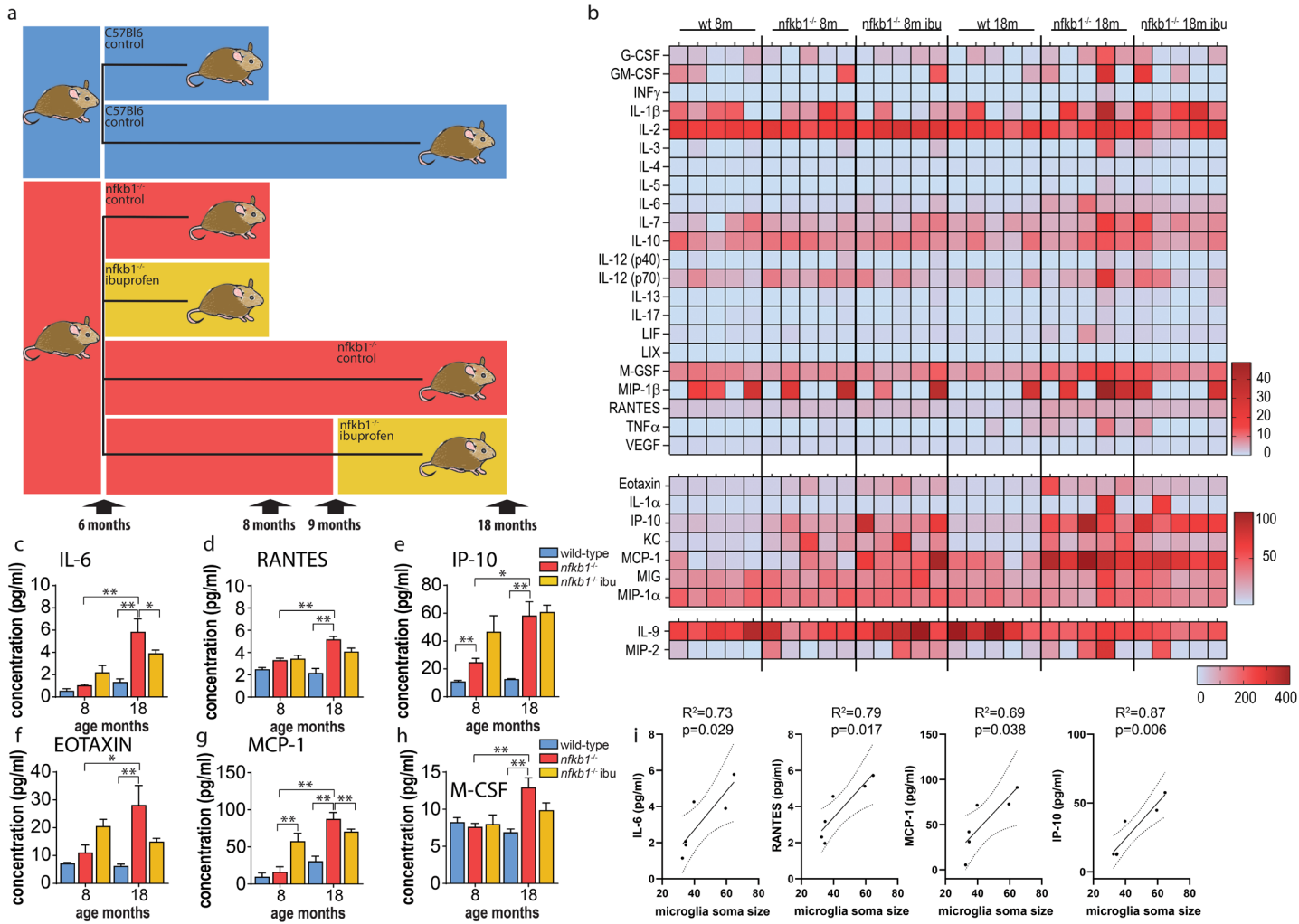

Supplementary Figure 2

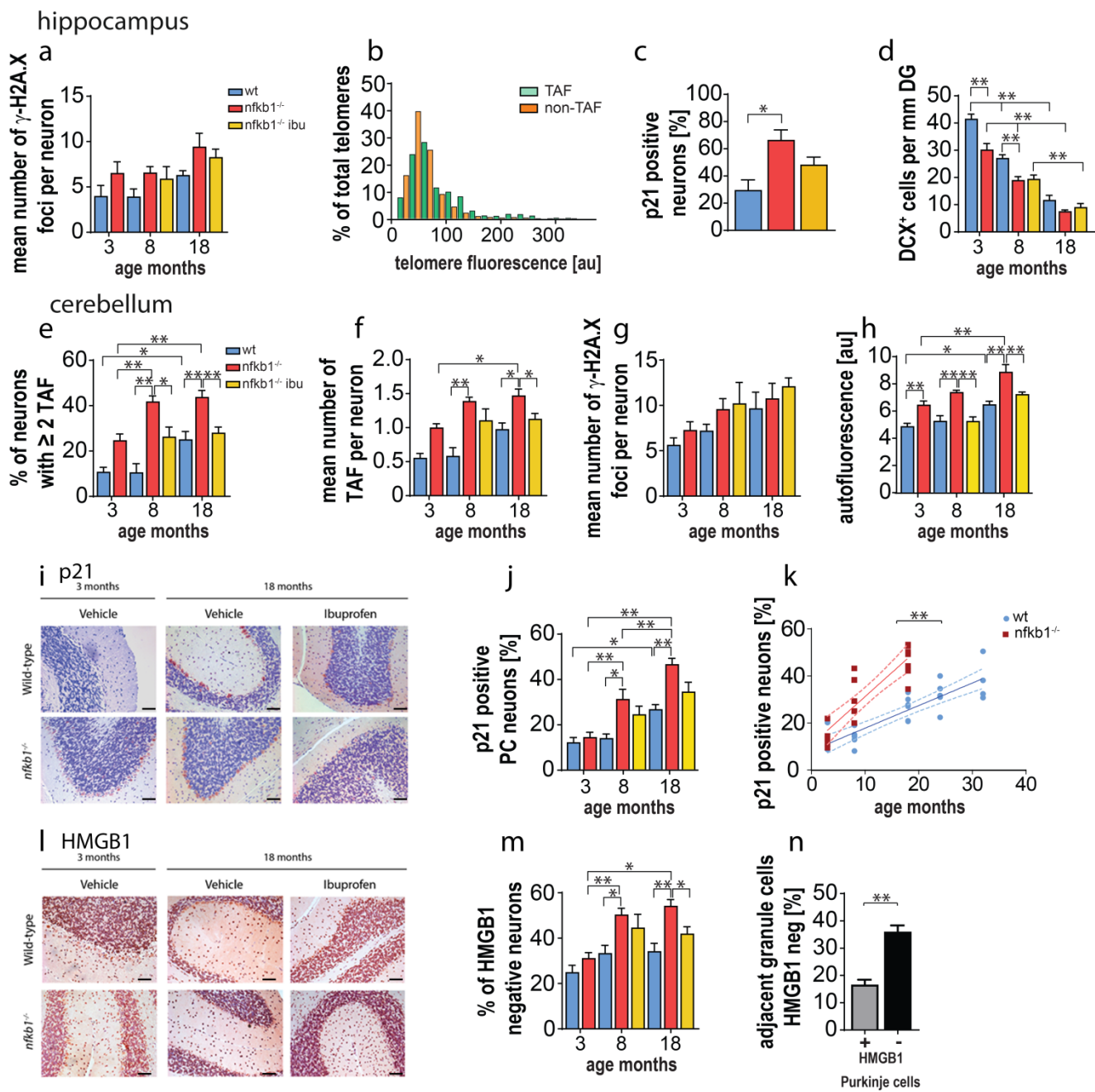

Supplementary Figure 3

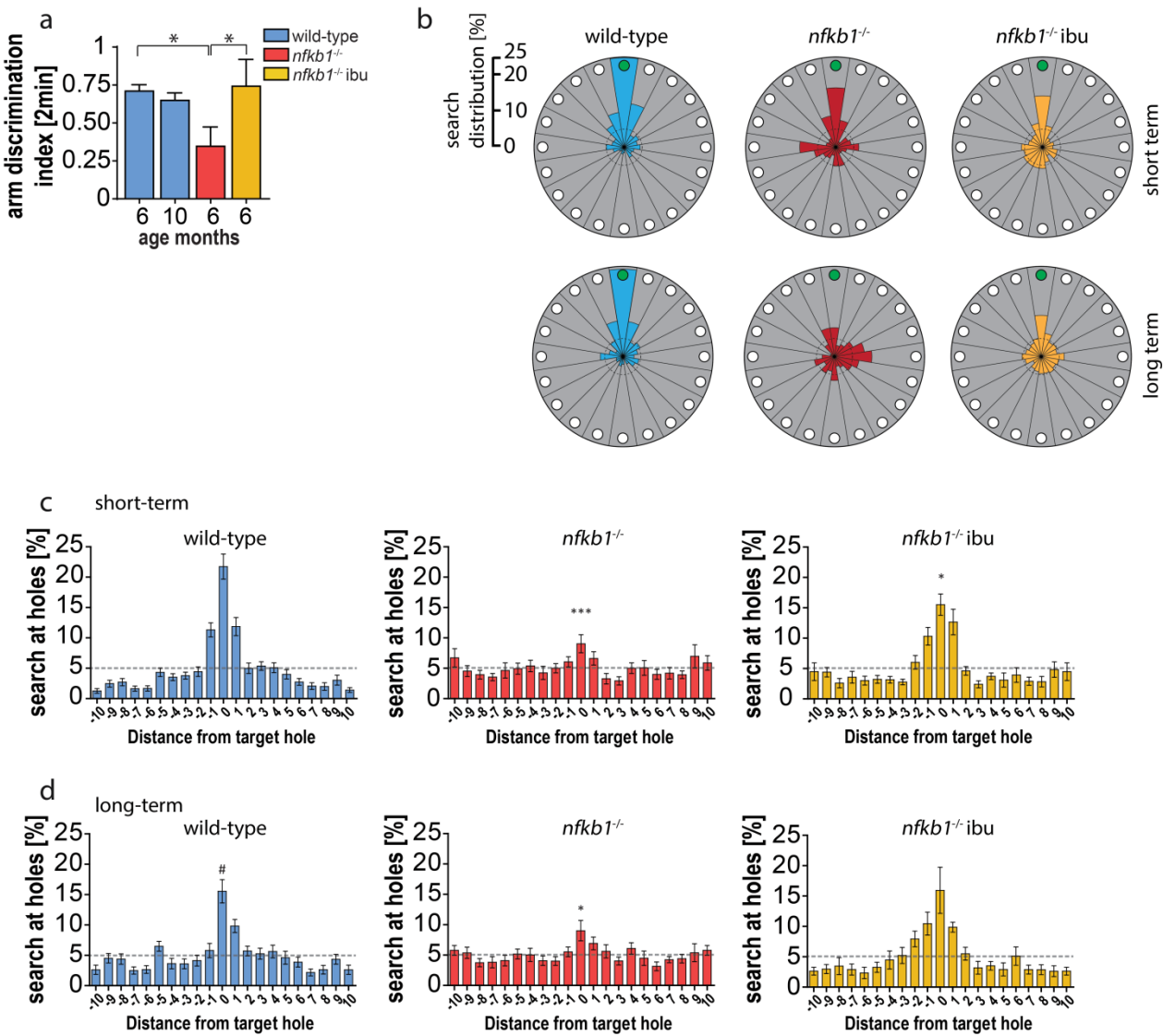

Supplement: Supplementary file 1 — Fig S1‐S3 [file ACEL-19-e13188-s001.pdf]
