# Supplementary material for: Anti‐inflammatory treatment rescues memory deficits during aging in nfkb1 −/− mice
Source: Aging Cell. 2020 Sep 11;19(10):e13188. doi: 10.1111/acel.13188 (PMC7576267; doi:10.1111/acel.13188)
Supplement: Supplementary file 2 — Fig S1‐S3‐Legends [file ACEL-19-e13188-s002.pdf]

Figure 1: Treatment with NSAID reduces neuro-inflammation in premature ageing mouse model

At 6 and 9 months of age male wild-type and *nfkb1*$^{-/-}$ mice were split into groups and assigned to control or treatment groups before harvested at 8 and 18 months of age, respectively (**see Supplementary Figure S1a**). **a)** Cytokine expression pattern in whole brain homogenates for 8 and 18 months old wild-type and *nfkb1*$^{-/-}$ mice treated with/without ibuprofen. Data shown in pg/ml. **b)** Micrographs showing iba-1 IHC staining in hippocampus of paraffin embedded brain sections in 8 and 18 months old animals in the indicated groups. Areas in dashed lines are shown magnified at the right, scale bars = 50μm. Frequencies of microglia per mm^2 hippocampus in **c)** CA3, **d)** CA1 and **e)** DG of wild-type and *nfkb1*$^{-/-}$ mice. **f)** Average soma size per microglia was assessed in CA3 of the hippocampus.

Significant differences (ONE WAY ANOVA) are indicated with * P<0.05 and ** P<0.001. n= 4-5 mice per group.

Figure 2: Chronic inflammation leads to neuronal senescence and can be ameliorated by NSAID treatment

a) Representative images showing γ-H2A.X (green) and telomere FISH (red) in NeuN (pink) positive cells of the indicated animals at 18 months of age. White arrows indicate Telomere Associated Foci (TAF) and area in white dash-lined quadrat is shown in magnification below. Scale bars = 20μm in magnified images = 10μm. **b)** Percentage of neurons with ≥2 TAF and **c)** mean number of TAF. **d)** Representative images showing HMGB1 IHC staining in CA3 of the hippocampus in 18 months old animals. Black arrows indicate neurons with loss of nuclear HMGB. Areas in dashed lines are shown magnified on right. Scale bars = 50μm. **e)** Percentage of neurons showing nuclear HMGB1 loss. **f)** Micrographs showing lipofuscin granules (autofluorescence) in hippocampal neurons. White arrows indicate granules and area in white dash-lined quadrat is shown in magnification below. Scale bars = 20μm. **g)** Graph showing mean number of autofluorescent granules per neuron in indicated mice and treatments.

Significant differences (ONE WAY ANOVA) are indicated with * P<0.05 and ** P<0.001. n= 4-7 mice per group.

Figure 3: Reduced spatial discrimination and memory in *nfkb1*$^{-/-}$ mice can be rescued by NSAID

a) Cartoon showing Y-maze set-up for training and trial period. **b)** Pie-charts depicting first choice of arm entry (blue=novel arm, grey=familiar arm) in Y-maze. **c)** Latency to enter novel arm. **d)** Visual depiction of search distribution around the Barnes maze in 18 months old animals (target hole in green at 12 o'clock position). Graphs showing **e)** average number of errors prior and **f)** latency to finding the target hole in 18 months old animals. **g)** Visual portrayal of search strategies in Barnes maze testing. Graphs displaying the percentage of each strategy used to find the target hole in **h)** 8 months and **i)** 18 months old animals.

Significant differences (ONE WAY ANOVA, **e-f** 2-WAY ANOVA) are indicated with * P<0.05 and ** P<0.001. n= 4-18 mice per group in Y-maze, 8-11 in Barnes maze.

Figure 4: Deficits in neuronal oscillation is improved after NSAID treatment

a) 1 sec example traces 30min after carbachol and **b)** at stable time-point **c, d)** show the corresponding power spectra for 60 sec traces at 30 and 3 hours post carbachol. **e)** Box plot shows gamma frequency area power of the oscillations over time after carbachol application. Slices were allowed one hour in the recording chamber (baseline + 1 hr) before the addition of carbachol. Graphs showing **f)** oscillation area and **g)** oscillation frequency area power at stable time-point. **h)** Representative auto-correlations of gamma oscillations recorded in CA3. **i)** Normalised rhythmicity index (RI) of gamma frequency oscillations in CA3.

Significant differences (TWO WAY REPEATED MEASURES for e-g and ONE WAY ANOVA for i) are indicated with * P<0.05 and ** P<0.001. n= 3-6 slices per mouse and 4-5 mice per group.

Supplementary Figure 1:

a) C57Bl6 and *nfkb1*$^{-/-}$ mice where treated as indicated. **a)** Cytokine array results shown per animal in pg/ml. Cytokines are separated in 3 heat maps based on expression values. **c-h)** Extracted data from cytokine array for **c)** IL-6, **d)** RANTES, **e)** IP-10, **f)** EOTAXIN, **g)** MCP-1 and h**)** M-CSF. **i)** Correlations between microglia activation (some size) in hippocampus and whole brain cytokine levels (n= 3, wt and nfkb1$^{-/-}$ mice at 18m).

Significant differences (TWO WAY ANOVA) are indicated with * P≤0.05 and ** P≤0.001. n= 5 mice per group.

Supplementary Figure 2:

Average number of **a)** γ-H2A.X foci per neuron in CA3 **b)** Telomere fluorescence intensity histograms divided in TAF (light green) and non-TAF (orange) in CA3 NeuN positive neurons from 3 wild-type 18 month old mice **c)** p21 positive neurons in CA3 at 18 months. **d)** Average numbers of DCX+ cells per mm length of Dentate Gyrus. **e) – n)** Senescence markers in Cerebellar Purkinje cells. **e)** Average number of Purkinje neurons with 2≥TAF in cerebellum. Mean number of **f)** TAF, **g)** γ-H2A.X foci and **h)** mean autofluorescent intensity per Purkinje neuron. **i)** Representative images of p21 IHC staining. Scale bars = 100μm **j)** Percentage of p21+ neurons in cerebellum. **k)** the frequency of p21+ neurons in cerebellum increases faster in *nfkb1*$^{-/-}$ (dark red) than wild-type (blue) mice with age. **l)** Representative images of HMGB1 IHC staining. Scale bars = 100μm **m)** Percentage of HMGB1negative Purkinje neurons in cerebellum and **n)** HMGB1 status in adjacent granule cells to HMGB1negative Purkinje neurons, from 3 wild-type 18 month old mice **c)** p21 positive neurons in CA3 at 18 months.

Significant differences (ONE WAY ANOVA, **b)** Mann-Whitney U k) t-test) are indicated with * P<0.05 and ** P<0.001. n= 3-8 mice per group.

Supplementary Figure 3:

a) Arm discrimination index in Y-maze testing in 6 and 10 months old animals. **b)** Visual depiction of search distribution around the Barnes maze in 8 months old animals. Graphs are showing the average numbers of head pokes in each hole of the Barnes maze for 18 months old animals **c)** in short-term and **d)** long-term assessments, with differences in frequency of searches at the target hole assessed.

Significant differences (ONE WAY ANOVA, c-d) 2-WAY ANOVA) are indicated with * P<0.05 and ** P<0.001. n= 4-18 mice per group.

Figure1

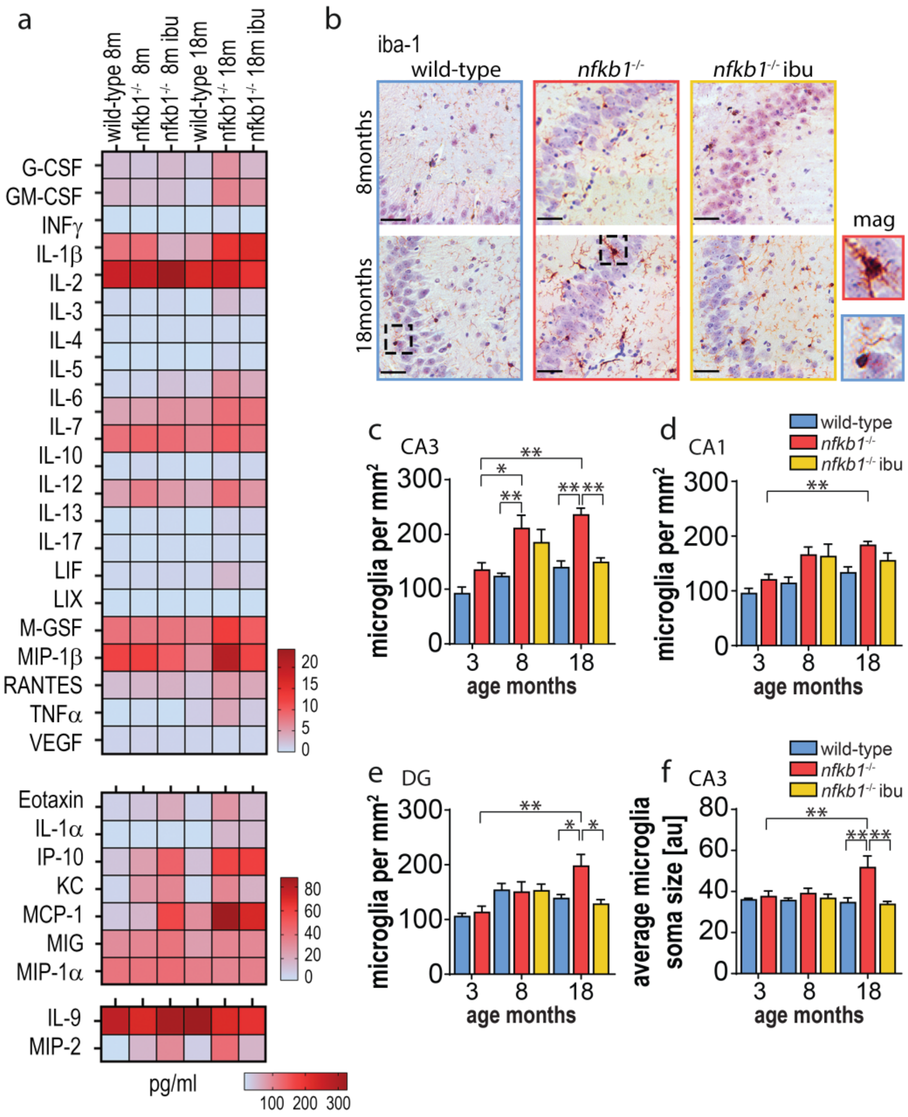
a
wild-type 8m
nfkb1-/- 8m
nfkb1-/- 8m ibu
wild-type 18m
nfkb1-/- 18m
nfkb1-/- 18m ibu
G-CSF
GM-CSF
INFγ
IL-1β
IL-2
IL-3
IL-4
IL-5
IL-6
IL-7
IL-10
IL-12
IL-13
IL-17
LIF
LIX
M-GSF
MIP-1β
RANTES
TNFα
VEGF
20 15 10 5 0
Eotaxin
IL-1α
IP-10
KC
MCP-1
MIG
MIP-1α
80 60 40 20 0
IL-9
MIP-2
pg/ml
100 200 300
b
iba-1
wild-type
nfkb1-/-
nfkb1-/- ibu
8months
18months
mag
c
CA3
microglia per mm²
age months
d
CA1
wild-type
nfkb1-/-
nfkb1-/- ibu
e
DG
f
CA3
average microglia soma size [au]
0 100 200 300
3 8 18
0 20 40 60 80

Figure2

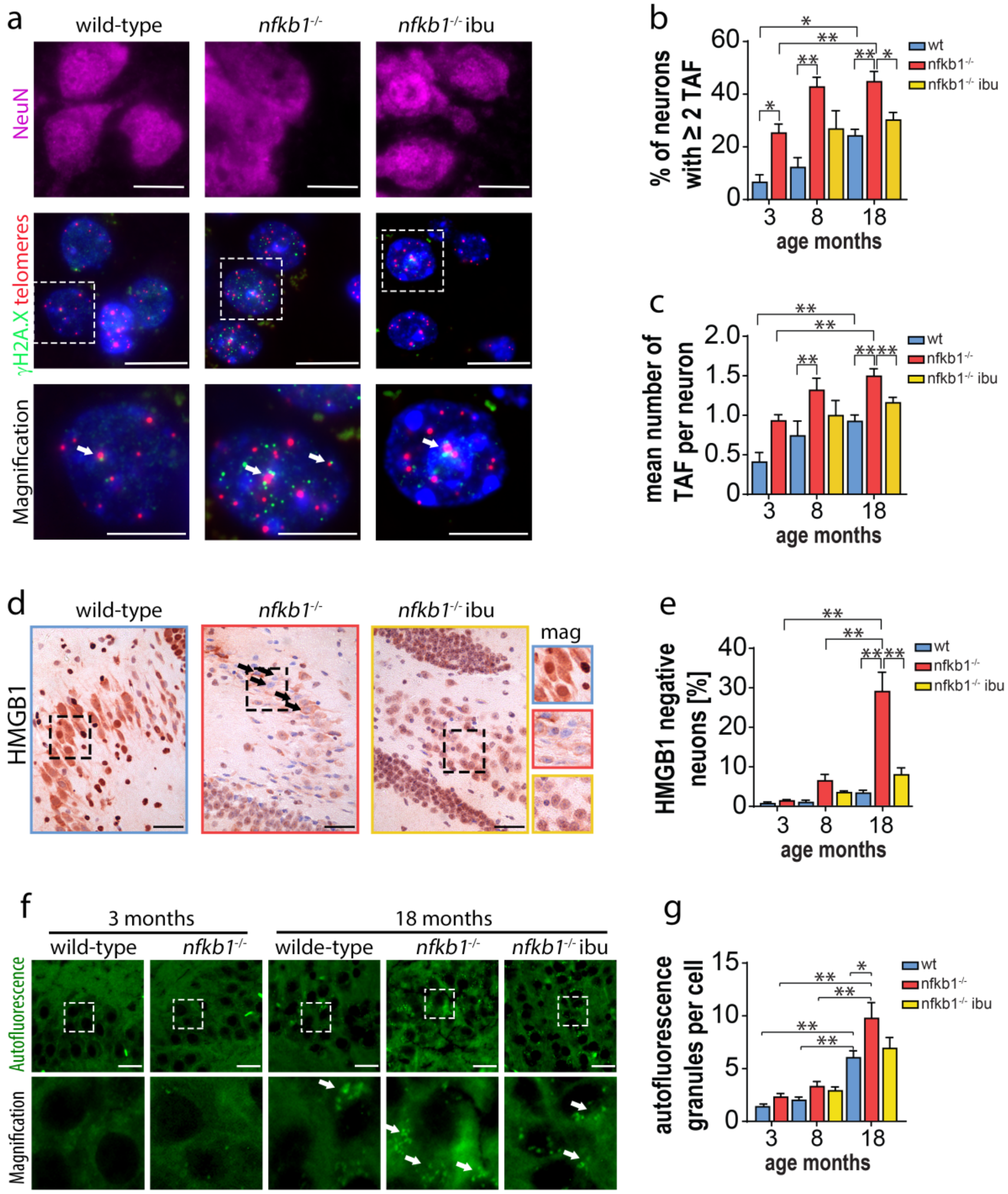
a
wild-type
nfkb1-/-
nfkb1-/- ibu
NeuN
γH2A.X telomeres
Magnification
b
% of neurons with ≥ 2 TAF
age months
wt
nfkb1-/-
nfkb1-/- ibu
c
mean number of TAF per neuron
age months
d
HMGB1
mag
e
HMGB1 negative neuons [%]
age months
f
3 months
18 months
wild-type
wilde-type
nfkb1-/- ibu
Autofluorescence
Magnification
g
autofluorescence granules per cell
age months

Figure 3

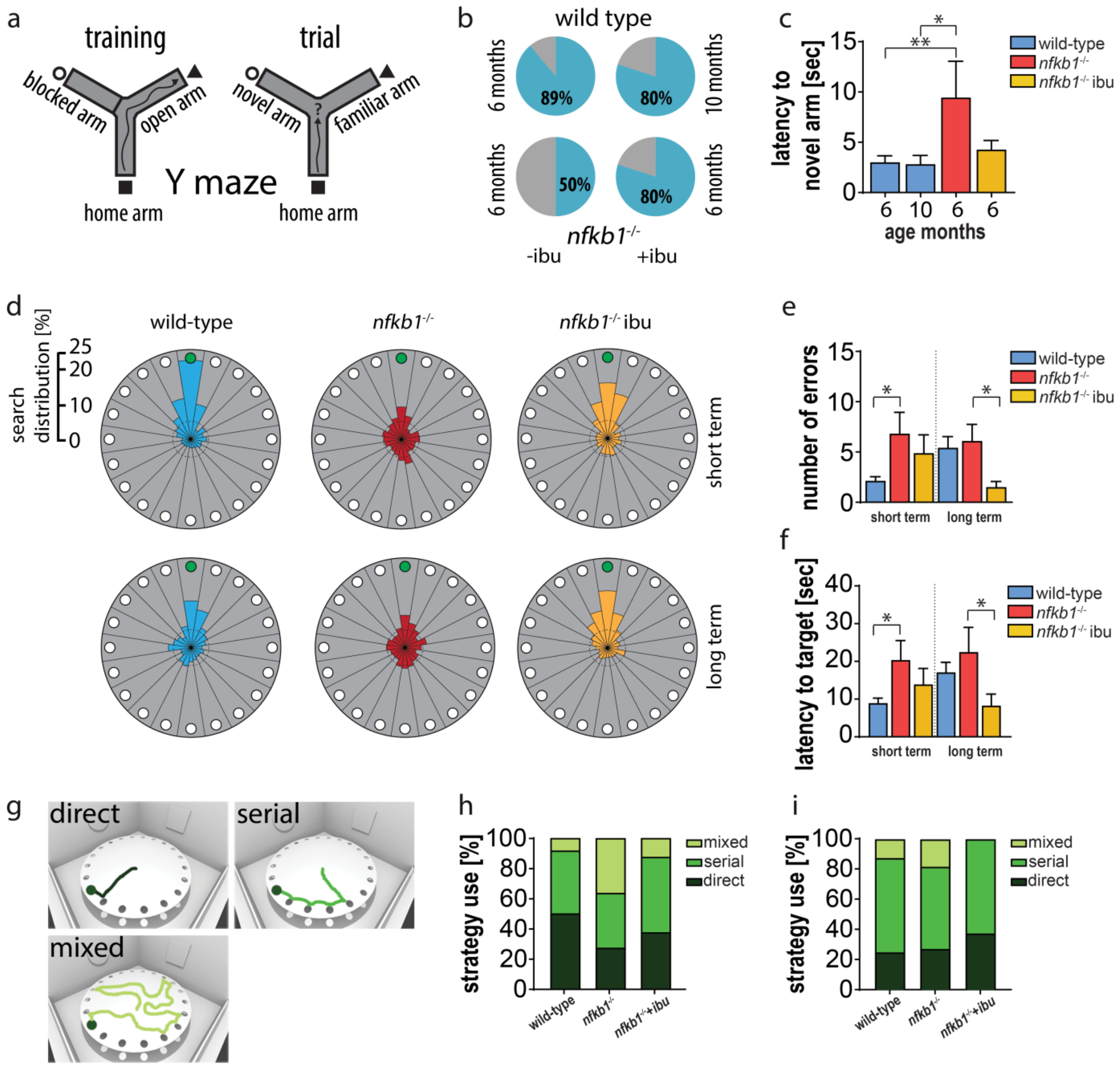
a
training
trial
blocked arm
open arm
novel arm
familiar arm
Y maze
home arm
home arm
b
wild type
6 months
10 months
89%
80%
6 months
6 months
50%
80%
nfkb1-/-
-ibu
+ibu
c
latency to novel arm [sec]
age months
wild-type
nfkb1-/-
nfkb1-/- ibu
d
search distribution [%]
wild-type
nfkb1-/-
nfkb1-/- ibu
short term
long term
e
number of errors
short term
long term
f
latency to target [sec]
short term
long term
g
direct
serial
mixed
h
strategy use [%]
mixed
serial
direct
wild-type
nfkb1-/-
nfkb1-/-+ibu
i
strategy use [%]
mixed
serial
direct
wild-type
nfkb1-/-
nfkb1-/-+ibu

Figure 4

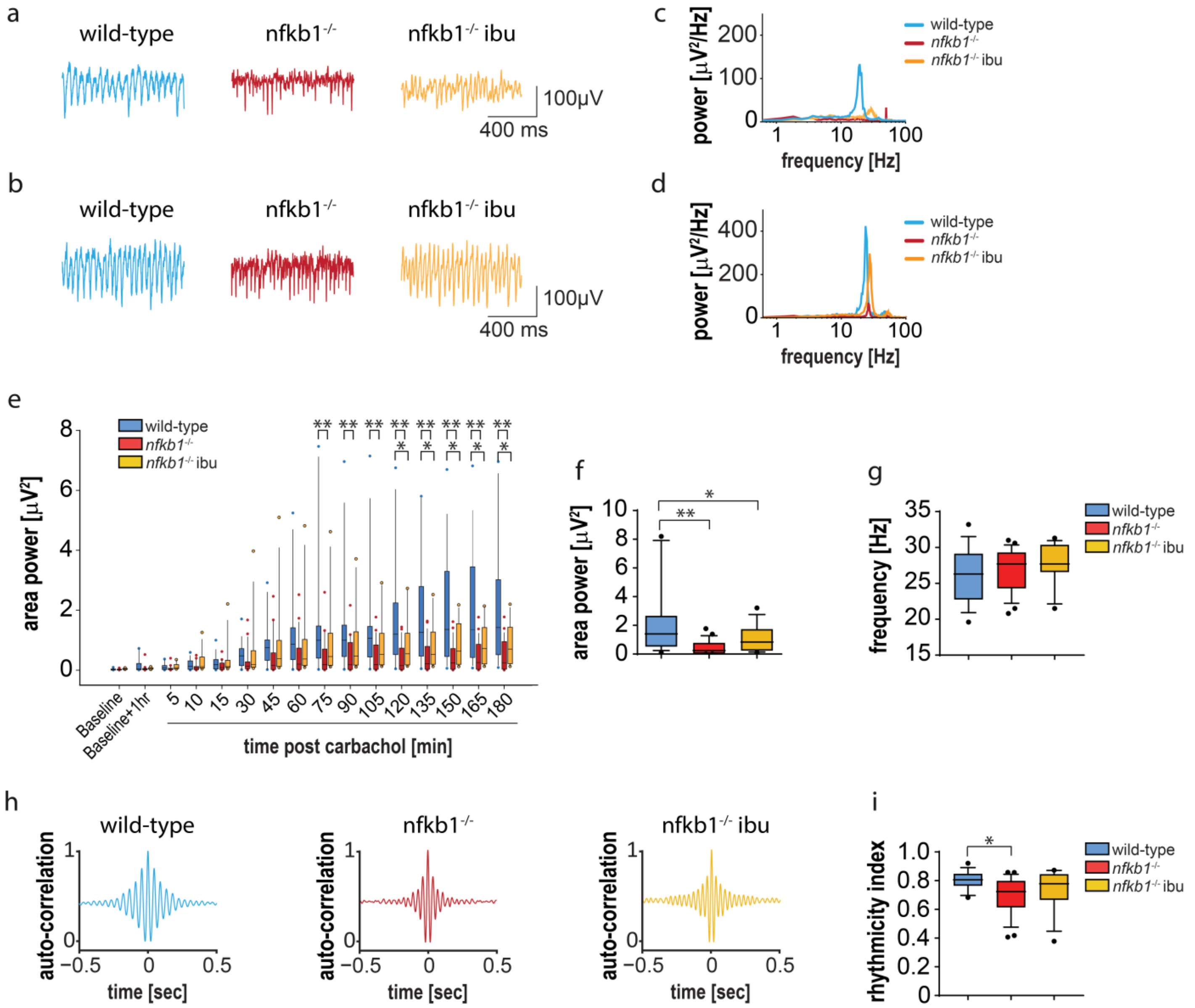
a
wild-type
nfkb1-/-
nfkb1-/- ibu
100µV
400 ms
b
wild-type
nfkb1-/-
nfkb1-/- ibu
100µV
400 ms
c
power [µV²/Hz]
frequency [Hz]
wild-type
nfkb1-/-
nfkb1-/- ibu
d
power [µV²/Hz]
frequency [Hz]
wild-type
nfkb1-/-
nfkb1-/- ibu
e
area power [µV²]
wild-type
nfkb1-/-
nfkb1-/- ibu
Baseline
Baseline+1hr
time post carbachol [min]
f
area power [µV²]
g
frequency [Hz]
wild-type
nfkb1-/-
nfkb1-/- ibu
h
wild-type
nfkb1-/-
nfkb1-/- ibu
auto-correlation
time [sec]
i
rhythmicity index
wild-type
nfkb1-/-
nfkb1-/- ibu
